# Carbon and Nitrogen Dynamics, and CO_2_ Efflux in the Calcareous Sandy Loam Soil Treated with Chemically Modified Organic Amendments

**DOI:** 10.3390/molecules26164707

**Published:** 2021-08-04

**Authors:** Ahmed Mohammed-Nour, Mohamed Al-Sewailem, Ahmed H. El-Naggar, Mohamed H. El-Saeid, Anwar A. Aly, Jamal Elfaki

**Affiliations:** 1 Department of Soil Science, College of Food and Agriculture Sciences, King Saud University, P.O. Box 2460, Riyadh 11451, Saudi Arabia; wdalkhatem@yahoo.com (A.M.-N.); sewailem@ksu.edu.sa (M.A.-S.); elsaeidm@ksu.edu.sa (M.H.E.-S.); AAALY@KSU.EDU.SA (A.A.A.); jemy20001@hotmail.com (J.E.); 2 Soil and Water Research Centre, Agricultural Research Corporation, P.O. Box 126, Wad-Medani 21111, Al Jazirah, Sudan; 3 Sustainable Natural Resources Management Section, International Centre for Biosaline Agriculture (ICBA), Dubai 14660, United Arab Emirates; 4 Department of Soil Sciences, Faculty of Agriculture, Ain Shams University, Cairo 11241, Egypt; 5 Soil and Water Science Department, Alexandria University, Alexandria 21545, Egypt; 6 Faculty of Agriculture, Nile Valley University, P.O. Box 1843, Atbara 45512, River Nile State, Sudan; 7 Desert Farming Techniques and Agricultural Biotechnology Programs, Sultan Qaboos Chair in Desert Farming, Department of Natural Resources & Environment, Arabian Gulf University, P.O. Box 26671, Manama 323, Bahrain

**Keywords:** organic wastes, cow manure, CO_2_ effluxes, ammonia stripping, C:N ratio

## Abstract

In Saudi Arabia, more than 335,000 tons of cow manure is produced every year from dairy farming. However, the produced cow manure is usually added to the agricultural soils as raw or composted manure; significant nitrogen losses occur during the storage, handling, and application of the raw manure. The recovery of ammonia from cow manure through thermochemical treatments is a promising technique to obtain concentrated nitrogen fertilizer and reducing nitrogen losses from raw manure. However, the byproduct effluents from the recovery process are characterized by different chemical properties from the original raw manure; thus, its impact as soil amendments on the soil carbon and nitrogen dynamics is unknown. Therefore, a 90-day incubation experiment was conducted to study the impact of these effluents on CO_2_ efflux, organic C, microbial biomass C, available NH_4_^+^, and NO_3_^−^ when added to agricultural soil. In addition to the two types of effluents (produced at pH 9 and pH 12), raw cow manure (CM), composted cow manure (CMC), cow manure biochar (CMB), and control were used for comparison. The application of CM resulted in a considerable increase in soil available nitrogen and CO_2_ efflux, compared to other treatments. Cow manure biochar showed the lowest CO_2_ efflux. Cumulative CO_2_ effluxes of cow manure effluents were lower than CM; this is possibly due to the relatively high C:N ratio of manure effluent. The content of P, Fe, Cu, Zn, and Mn decreased as incubation time increased. Soil microbial biomass C for soil treated with cow manure effluents (pH 12 and 7) was significantly higher than the rest of the soil amendments and control.

## 1. Introduction

Agriculture is the primary source of ammonia emission globally [1] and regionally in, e.g., Europe [2] and the USA [3]. Ammonia and its inorganic derivatives, nitrite, and nitrate are easily percolated to the ground and surface water, resulting in a deterioration of water quality and risk hazards in drinking water [4].

Regarding CO_2_ efflux from soil treated with organic amendments [5] showed that the total annual emissions of greenhouse gases (GHGs) from agriculture to the atmosphere in 2011 were 5335 Mt CO_2_ eq. Moreover, 25% of this amount is released into the atmosphere due to manure storage, management, and amendments. Soil CO_2_ efflux, as described by Kuzyakov [6] is efflux that comes from the root and rhizomicrobial respiration, decomposition of plant residues, the priming effect induced by root exudation or by addition of plant residues, and basal respiration by microbial decomposition of soil organic matter (SOM). Many vital factors control the CO_2_ efflux from the soil, such as climatic factors, temperature, water content [7], clay content [5], water holding capacity [8], and C:N ratio [9]. Risse et al. [10] reported that among many applied fertilizers to maize in China, CO_2_ and NO_2_ emissions were significantly correlated to pig manure and inorganic fertilizer due to their low C:N ratio. An incubation experiment [11] showed that the biochar amendments to soils reduced N-gases volatilization and decreased CO_2_ emissions from soils due to the low C:N ratio in the used biochar.

Therefore, several pretreatments have been suggested to increase the stability of N-rich wastes such as manure and produce organic amendments with a balanced C:N ratio and higher soil carbon stability. These methods include composting [12], pyrolysis process to produce biochar [13], and ammonia recovery from manure [14].

Composting animal manure and nitrogen-rich wastes is a technique to reduce nitrogen release from organic materials to soils. During composting, the C/N of mixed organic materials decreases due to the biochemical oxidation of organic matter [15]. For example, composting of “struvite” food waster reduced N loss by 18%, compared to raw wastes [16]. In a lysimeter study, it was found that the application of poultry manure and paper mill sludge blends resulted in a pulse of NO_3_-N (170 and 156mg N/L) that occurred three months following application. At the same time, compost treatments showed no such pulse [17]. Another study [18] found that composting of cattle manure doubled the soil humic substances, compared with the raw manure. They concluded that composting of cattle manure resulted in more stable and less decomposable organic compounds in soils. On the other hand, during the composting process, an appreciable amount of nitrogen is lost through the volatilization of ammonia [19].

Another method for increasing the C/N of the organic material is the pyrolysis of wastes to produce biochar. The pyrolysis process involves heating plant biomass in the absence of oxygen gas [20]. The pyrolysis process increased carbon percentage due to the increasing degree of carbonization. However, hydrogen and oxygen contents tend to decline [21]. Tomczyk et al. [22] found that during the pyrolysis of different feedstock to produce biochar, fixed carbon significantly increased. The N recovery was negatively correlated to a pyrolysis temperature.

The conversion of plant residues to biochar is an attractive strategy for mitigating atmospheric carbon dioxide emissions and enhancing carbon storage in soil [23].

Sigurnjak et al. [24] recently showed that nitrogen recovered from wastes could be used as a nitrogen fertilizer for crop cultivation. Mohammed-Nour et al. [14] achieved a 90% recovery of ammonia in cow manure through alkalization and thermal treatment. They suggested using the ammonia-stripping technique to reduce the environmental risks associated with ammonium volatilization from manure. However, the chemical properties of the produced effluents from the ammonia recovery process are significantly different from the raw manure. Therefore, its chemical behavior as a soil amendment is not known.

Stable soil organic carbon is one of the parameters used to evaluate the sustainability and efficiency of soil carbon sequestration. Jindo et al. [25] mentioned that different carbon fractions, such as the total organic carbon, water-soluble carbon, and microbial biomass carbon, were increased in amended soils, compared to the control. Additionally, Lima et al. [26] stated an increase in lignin and lignin-like products in the soil amended with compost.

The study’s primary objectives were to investigate the effects of cow manure-stripped ammonia effluents (CMSAEs) on temporal changes in soil CO_2_ flux and nitrogen forms (NH_4_^+^, NO_3_^−^, and total N), and to estimate the microbial biomass C as an indicator for microbial activity during incubation.

## 2. Materials and Methods

### 2.1. Soil and Organic Materials Characterization

#### 2.1.1. Soil

Topsoil (0–30 cm) was collected from an agricultural field located in Aloyyna, Riyadh, Saudi Arabia (24°54′27.36″ N and 46°23′35.49″ E). The sampled soils were homogenized and then sieved to less than 2 mm. Sieved soils were air-dried, and the physicochemical characterization was run according to standard methods [27]. pH was determined in 1:5 water extract (*w*/*v*) using pH meter, Orion Star A211. Electrical conductivity (EC) was measured in the filtrated extracts using YSI (Yellow Springs, OH, USA).

#### 2.1.2. Soil Amendments

##### Cow Manure (CM)

Cow manure (CM) was collected from the ALSAFI dairy farm situated in Al-Kharj, Saudi Arabia.

##### Cow Manure-Stripped Ammonia Effluents (CMSAEs)

CMSAEs are the byproduct of ammonia recovery from cow manure through the alkalization and distillation process [14]. In this experiment, two effluents from different alkalization degrees (pH 9 and 12) were studied.

Effluents were prepared by treating 100 g of fresh CM with either 2.44 or 0.5mL of 15 N KOH to bring pH to 12 or 9. Then, 100 mL of deionized water was added to manure paste in a one-lit round glass bottle. Mixtures were heated up to 95 °C for 5h. Ammonia was recovered in (25 mL of 0.5 N) sulfuric acid. CMSAEs were collected and then slowly cooled down to 25 °C.

##### Composted Cow Manure (CMC)

Composted cow manure (CMC) was obtained from (ALSAFI dairy products facility, Riyadh, Saudi Arabia); CMC was stored at 4 °C.

##### Cow Manure Biochar (CMB)

Briefly, 1kg of dry cow manure (CM) was pyrolyzed at 400 °C for 4 h. Cow manure biochar (CMB) was produced according to [21].

CMC and CMB were used in the incubation experiment as reference material to evaluate the C stability from CMSAEs of CM. Before application time, all organic materials were air-dried, grained, and sieved to 1 mm.

### 2.2. Chemical Analysis of Soil Amendments

Total nitrogen and carbon were determined using a EuroVector Elemental Analyzer EA3000 equipped with Callidus software SW v.5.1 (EuroVectorSpA, Milan, Italy). To determine total phosphorus, potassium, calcium, magnesium, and other micronutrients in manure, 0.2 g of dried manure were treated with 10 mL of concentrated nitric acid and digested according to the procedure of [28] using microwave digestion (MARS, CEM Corporation, Matthews, NC USA). The total concentration was determined in the digestate using an inductively coupled plasma optical emission spectrophotometer (ICP-OES, PerkinElmer Optima 4300 DV, Waltham, MA, USA).

### 2.3. Incubation Experiment

A long-term incubation experiment was carried out under laboratory conditions for 90 days. The experiment consists of five organic materials: CM, CMC, CMB, CMSAE (pH 12), and CMSAE (pH 9) plus the control. CMC and CMB were used to compare C and N dynamics with that of CMSAEs. In total, 100 g of 2 mm sieved calcareous soil (texture: sandy loam) was placed into 250 mL jars. Experimental soil was pre-incubated for 15 days at 25 °C to allow the soil to equilibrate after sieving and handling. The moisture of the soil samples was initially adjusted to 75% of the water holding capacity (WHC) through the addition of deionized H_2_O at regular intervals (1 to 2 weeks). The WHC was determined by saturating a sample of soil in filter paper placed in a glass funnel. Then, the water was drained for 2 h before the gravimetric soil moisture content (for 100% WHC) was determined by drying for 24 h at 105 °C. Amendments were added to the soil at a 50 mg C.g^−1^ soil based on the elemental C analysis of the used amendments—total added N amendments estimated before the experiment. A blank without N and C addition was used as a control. The jars were fastened airtight and incubated in a growth chamber at 30 °C. The samples’ moisture was periodically adjusted to the value of field capacity (27% *v*/*w*). The CO_2_ efflux from soil was measured at 1, 2, 4, 5, 10, 13, 18, 30, 40, 50, 60, 70, 80, and 90 days, using 10 mL of 1N NaOH solutions as the captured solution. At all sampling time, 10 g soil was extracted with 50 mL of 0.5 M K_2_SO_4_ before and after fumigation with chloroform. The extracts were titrated with Fe (NH_4_)_2_(SO_4_) 0.2 N to determine the amount of microbial biomass carbon during the 90-day incubation period, according to Anderson and Domsch, 1978. For available nitrogen determinations, 10 g of fresh soil were extracted with 2 M KCl and distilled with the Kjeldahl instrument; the nitrogen was received at 3% boric acid. Standard H_2_SO_4_ 0.01 N was used to titrate boric acid in order to obtain mineral nitrogen content (NH_4_^+^ and NO_3_^−^); for pH and EC determination, suspension of (1:5) soil: water was made, and Orion Star (A211) pH meter was used to determine soil pH. Electrical conductivity (EC) was measured in the filtrated extracts using an EC meter (YSI, USA). Finally, for available phosphorus and micronutrients, an extract of 0.005 M ammonium bicarbonate (DTPA) was prepared, and the inductively coupled plasma optical emission spectrophotometer (ICP-OES, PerkinElmer Optima 4300 DV, USA) was used to estimate the available concentration of extracting solution.

### 2.4. Statistical Analysis and Experimental Design

The presented data are averages of three replicates. The measured soil chemical properties, carbon efflux rate, and microbial carbon were statistically compared using Duncan’s multiple range tests. Nutrient release from different organic amendments at different time intervals was statistically analyzed by completely randomized design under two (days of incubation and organic amendment) factorial arrangement. Statistical analysis of the data and simple correlation and regression analyses were performed using SPSS 19.0 software. The significance test was conducted at a 5 and 1% level of significance (*p*  ≤  0.05 and *p*  ≤  0.01).

## 3. Results and Discussion

### 3.1. Experimental Soil

The soil used in this study was agricultural soil with sandy loam texture, pH of 8.5, electrical conductivity of 0.6 dS m^−1^, total carbon 4.8%, and total nitrogen (N) content of 0.1%; calcium carbonate content was 29.9%.

#### 3.1.1. Soil pH

**Figure 1:**
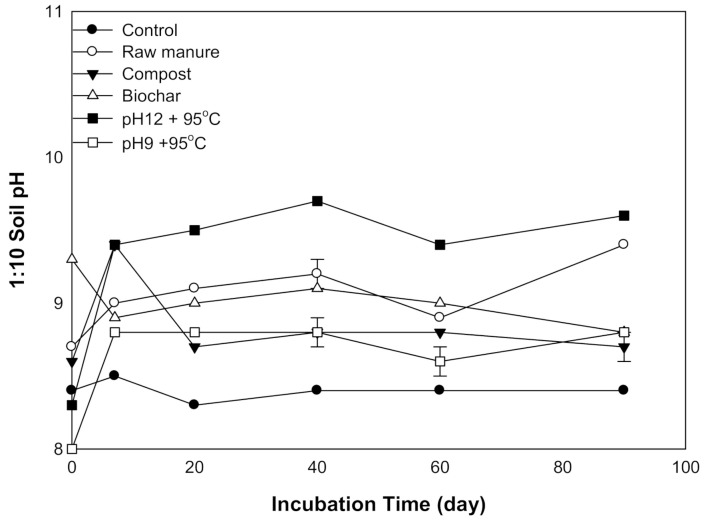
Variation in soil pH treated with different organic amendments derived from cow manure at different incubation times.

There were significant differences (*p* ≤ 0.01) in soil pH among the organic amendments and incubation time. Almost all treatments increased soil pH after seven days of incubation except for CMB (Figure 1). The highest pH value was 9.7, obtained by CMSAE (pH12), while the lowest pH was 8, obtained by CMSAE (pH 9). The soil pH of CM, CMC, CMB, and CMSAE (pH 9) was lower at start, Soil pH increased during the first 7 days of incubation, then, the pH become stable afterward. Higher pH values of CMSAE (pH 12) could be explained by the addition of more KOH before ammonia stripping, compared to CMSAE (pH 9). Initially, the CMB had the highest pH. The increase of basic metals in the biochar might be the reason to raise soil pH after biochar application [29]. It was reported reported a 0.8- to 0.1-unit rise in the soil pH as a result of biochar application to the soil [30].

Soil pH after 20 days tends to increase and then fall at 60 days, except for CMC, which depressed earlier at 20 days incubation time. This was also reported by [31]: they stated that the cation exchange capacity increases as a result of organic matter decomposition, therefore increasing the soil buffering capacity. The high buffering capacity of the soil increases by the increasing decomposition of organic material, releasing OH^−^ and CO_2_ and thus increasing pH. The lack of effect of the organic materials on soil pH may most likely be confirmed by the findings of [32]. At 90 days, pH tends to equilibrate, while row caw manure and CMSAE (pH 12) tend to increase. The second pH increase could be attributed to the breakdown of organic matter, which results in H^+^ ions being released into the soil from the functional groups, which led to pH decrease [33].

#### 3.1.2. Soil EC

**Figure 2:**
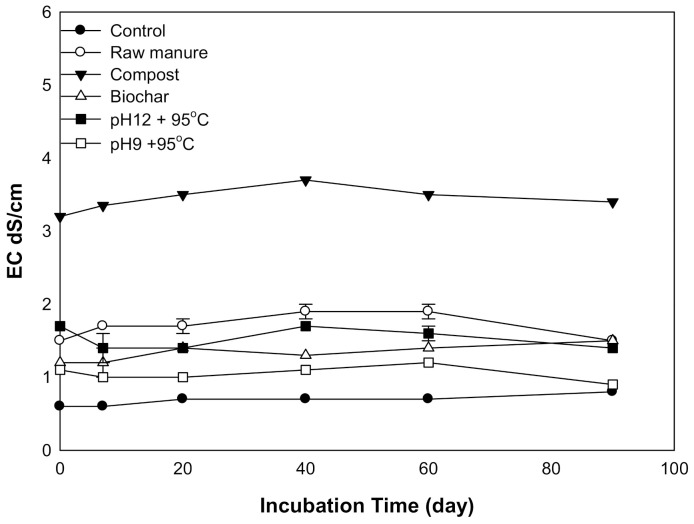
Variation in soil EC affected by different organic amendments derived from cow manure at different incubation times.

There was a statistically significant difference in soil EC due to the addition of the different amendments. The incubation time did not affect soil EC. The application of various organic amendments resulted in an initial rise in soil EC, followed by a steady EC afterward. All amendments to EC were less than 2 dS m^−1^ except for CMC, which was 3 dS m^−1^ (Figure 2). The soil incubated with CMC showed the highest EC. The soil EC of CMB was increased during 0–40 days. The CMSAEs showed less EC, as compared to CMC, but similar to that of the CMB. Higher CM soil EC related to initial EC. The increase of soil salinity due to the application of cow manure was reported by [34]. They considered manure as an essential source of soil salinity. The same observation was reported by [35], who found that huge manure quantities induced soil salinity. These quantities increased salt content and soil EC. CM can increase salinity due to the presence of water-soluble nutrients such as ammonium, Na, Ca, Mg, K, Cl, SO_4_, and HCO_3_, as well as the use of nutritional salts (NaCl).

#### 3.1.3. Available Phosphorus

**Figure 3:**
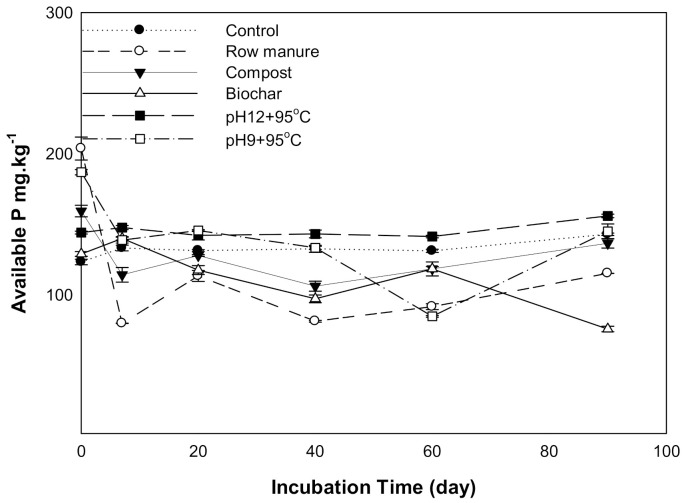
Available phosphorus affected by different organic amendments derived from cow manure at different incubation times.

The results for available phosphorus (P) affected by different organic amendments derived from CM are shown in Figure 3. There was a statistically significant difference in available P as a result of organic amendments and incubation time. The available P in soils treated with CM, CMC, and CMSAE (pH 12) decreased between 0 to 7 days and then tended to increase as the incubation time increased. This decrease may result from CaCO_3_, which has been reported to retain P as dicalcium phosphate and octacalcium phosphate [36]. Soil treated with CMSAE (pH 12) showed the highest available P between days 20 to 90. This increase in soil available P might be due to the high humic acid content in manure effluents, compared to CM and CMB, as found by [37]. They indicated that the presence of humic acid slows the precipitation of poorly soluble Ca phosphates.

P adsorption and desorption by biochar and other carbonaceous materials are governed mainly by soil pH [38]. In addition, P availability in soil is controlled by Ca and Mg ions under high pH conditions. Thus, the decline in the availability of P mainly qualified to the high content of CaCO_3_ and high soil pH by forming less soluble compounds.

#### 3.1.4. Available Iron

**Figure 4:**
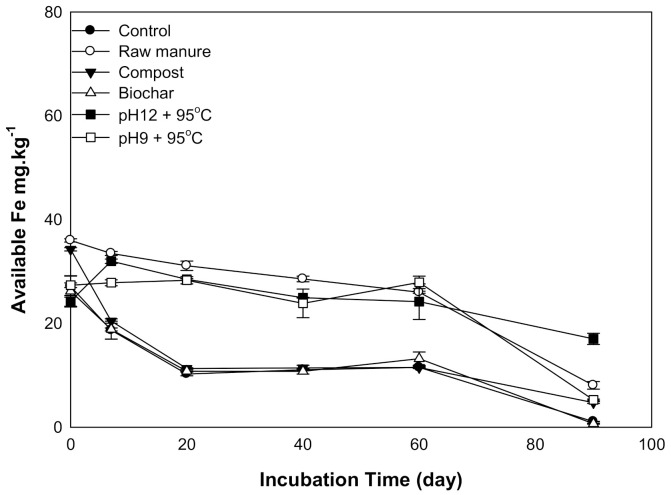
Soil available iron affected by different organic amendments derived from cow manure at different incubation times.

There was a significant difference in soil available Fe as a result of the addition of organic amendments. The Fe declined as incubation time decreased (Figure 4). The Fe content values are plotted in two areas: sharp Fe decrease, in CMC and CMB, and gradual Fe decrease, in CMSAEs and CM. After 60 days, Fe content depleted to the lowest value for all treatments. The Fe content of soils treated with CM and CMSAEs was higher, compared to other amendments. This may be related to the high Fe content of CM, and CMASEs had high humic substance content that may contribute to Fe availability via the formation of water-soluble Fe–humic substance complexes, which quickly move in the soil [39]. Uptake of 59Fe from 59Fecomplex has been measured even at pH values compatible with those found in calcareous soils [40]. However, calcareous soils tend to reduce Fe availability due to high pH in this soil [41,42]. The high pH increases the hydroxyl functional group, and subsequently, Fe hydroxide precipitates [43].

#### 3.1.5. Available Copper

**Figure 5:**
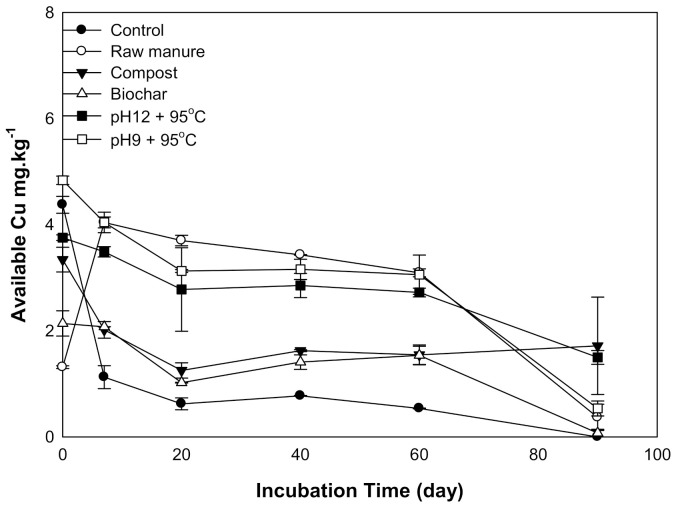
Soil available copper, affected by different organic amendments derived from cow manure at different incubation times.

There was a significant difference in soil Cu availability as a result of organic amendments. The available Cu declined with increasing incubation time. The soil amendments could be divided into two groups according to soil available Cu curves: Group one includes CMC and CMB and is characterized by a sharp decrease in available Cu, while group two has CM and CMSAEs and is characterized by a gradual reduction of available Cu. Between 0 to 20 days, Cu decreased in all amendments except CM; afterward, available Cu was almost stable until 60 days, and then its content decreased at 90 days (Figure 5). The high available Cu at the beginning is explained by low soil pH, as shown in Figure 1. On the other hand, the available Cu of CMSAE-treated soils was higher than that of CMC and CMB; this might be due to the temperature of CMSAEs preparation, which promotes the degradation of Cu binding compounds, consequently producing more Cu in soil solution [44].

#### 3.1.6. Available Zinc

**Figure 6:**
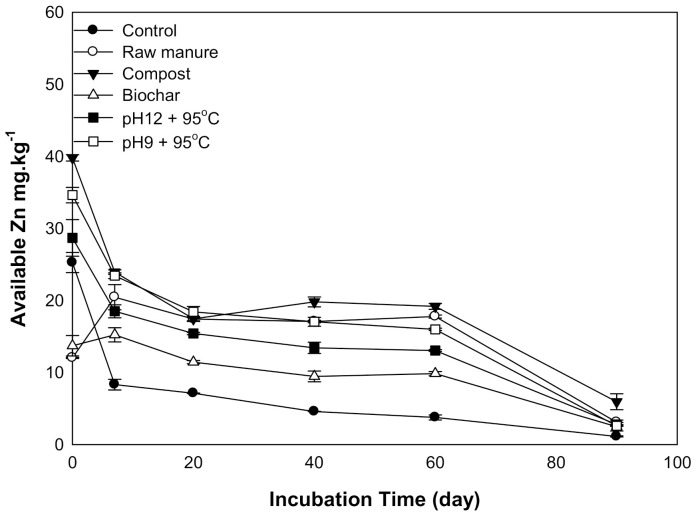
Soil available Zinc affected by different organic amendments derived from cow manure at different incubation times.

There were significant differences in Zn content was observed as a result of organic amendments’ application. Zn content generally declined with increasing incubation time (Figure 6). The highest recorded Zn content was (39.7 mg kg^−1^) for CMC treatment, while the lowest Zn content was (1.1 mg kg^−1^) in control soils after 90 days. Higher soil pH and CO_3_ content negatively affected Zn content and decreased its availability. Smith [45] showed that aerobic composting processes increased heavy metals’ stability through the formation of complexes with organic matter.

High pH values increase soil hydroxyl functional groups, and subsequently, zinc hydroxide and zinc carbonate are expected to be precipitate and reduce Zn availably [43].

#### 3.1.7. Available Manganese

**Figure 7:**
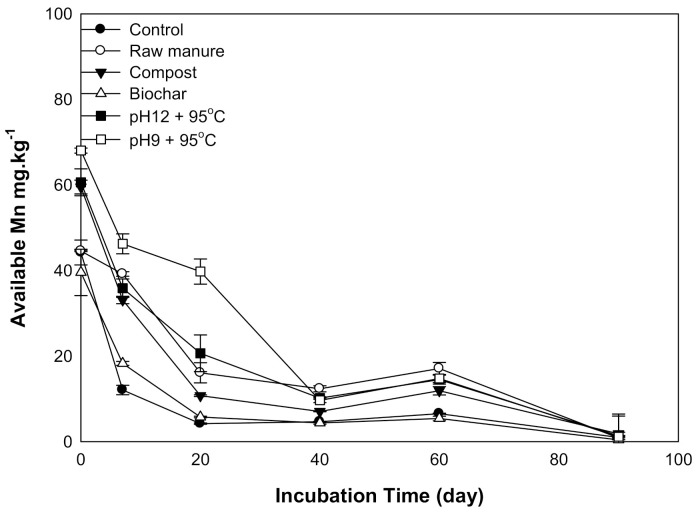
Soil available Mn affected by different organic amendments derived from cow manure at different incubation times.

Higher Mn content was observed at the start of the incubation. Afterward, Mn concentration sharply decreased for all organic amendments in 20 days, except for a gradual decline for CMSAE (pH 9), which reached 9.6 mg kg^−1^ at 40 days. The highest Mn content was 86 mg kg^−1^, observed at CMSAE (pH 9), while the lowest available Mn concentration (0.38 mg kg^−1^) was observed in the case of CMB-treated soils at day 90. Available Mn was significantly reduced in CMB, compared to CMSAE (pH 9), at seven days incubation time. At the same time, its content decreased by 1:8 ratio at 20 days (Figure 7). Low Mn content may have resulted from the mass losses after the decomposition of organic matter [46]. Similar results were also obtained by Zeng et al. [43] who found the Mn content was below the critical level when soil pH was increased.

### 3.2. Nitrogen Dynamic

#### 3.2.1. Available Nitrate

**Figure 8:**
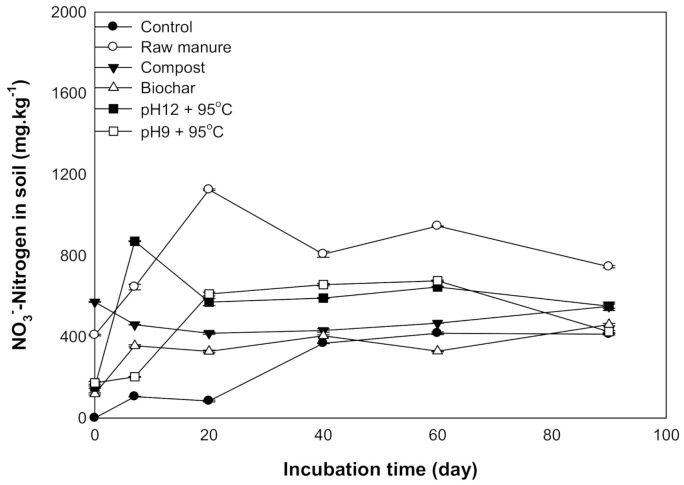
Available NO_3_^−^mg kg^−1^ affected by different organic amendments derived from cow manure at different incubation times.

Nitrate nitrogen is considered one of the measurements for soil nitrogen availability. The results showed that the addition of different organic materials caused an increase in the KCl extract NO_3_^−^ of nitrogen concentrations as incubation time increased. The maximum nitrification was recorded at 60 and 90 days after incubation. The maximum NO_3_^−^ ranged between 1124 ±4.2 and 870 ± 2 mg kg^−1^ soil for CM and CMSAE (pH 12), respectively (Figure 8).

Generally, incubation time increased NO_3_^−^ content. Increase NO_3_^−^ content after 20 days might be related to biological oxidation of ammonia to nitrate through nitrification process, according to [47] and [48] as follows:

2NH_4_^+^ + 3O_2_= 2HNO_2_ + 2H^+^ + 2H_2_O (Nitrosomas sp)
2HNO_2_+ O_2_ = 2NO_3_^−^ + 2H^+^ (Nitrobacter sp)

Environmental factors that affect ammonification and nitrification are temperature, water status, and C:N ratio [49,50]. Ammonium nitrification could be a reason for the increase in NO_3_^−^ the content of soil incubated with an organic compound. The rise in nitrification at 60 days was also found by [51], who stated that the addition of poultry manure to soil resulted in NO_3_^−^ accumulation at 60 says. This was mainly due to the increased activity of nitrifying bacteria. These findings agree with those results found by [52], who found that the ammonium content extracted from soil treated with fresh poultry litter declined during the first 30 days of incubation, and there was a rapid increase in NO_3_^−^ contents.

Additionally, high soil pH could be a reason for the inhibition of nitrifying bacteria growth, resulting in incremental NO_3_ content [53]. It is essential to highlight the importance of NO_3_^−^ management while applying cow manure to prevent NO_3_^−^ peculation through the soil profile. The CMC and CMSAE (pH 12) showed the highest NO_3_ at incubation. This increase is due to the appreciable content of NO_3_^−^ in CMC. Paul and Beauchamp [54] found that the soil amended with CMC contains more than 206 mg N per kg soil, compared to fresh CM. Hence, if CM is added to the soil under field conditions, we suggest that the best management practice would be to avoid the environmental risk of NO_3_-N leaching.

#### 3.2.2. Available Ammonium

Ammonium nitrogen is considered one of the measurements for soil nitrogen availability to plants. The results showed that the addition of different organic materials caused a significant difference in extracted NH_4_^+^ nitrogen content. Directly after amendments incorporation, the available ammonium increased with a relatively high rate, as incubation time increased, the NH^+^ release rate tended to decrease; these results point out the added manures increased available NH_4_^+^.Extracted ammonium increased immediately after manure applications, which was also found by [55], who reported a concentration of 1100 mg kg^−1^ ammonium following manure application. Microbes degrade simple organic compounds such as simple carbohydrates and amino acids in a short time. Therefore, soil available ammonium increases [56].

Over time, ammonium content decreased up to 60 days. This reduction was mainly due to the nitrification process through which ammonium is converted to NO_3_^−^, as stated by [57]. The NH_4_ content was high as a result of CM addition, as shown by [58], which occurred in the same manner when organic matter was added to cultivated soil. This may be due to dissolved organic matter, which increased the ammonium content [59]. These findings are consistent with those of [53]; they found that the NH_4_-N concentration extracted from soil treated with fresh manure declined during the first 30 days of incubation, but that there was a rapid increase in NO3-N concentrations. NH_4_-N immobilization by soil microbes and/or N losses such as ammonia volatilization might also be responsible for the rapid decrease in net N mineralization after 20 days of incubation [60]. The reduction in ammonium content may also be attributed to wide C/N, as stated by [61]. Additionally, [62] noted that the retention of ammonium into the negative charge of clay might be attributed to lower NH_4_^+^content.

**Figure 9:**
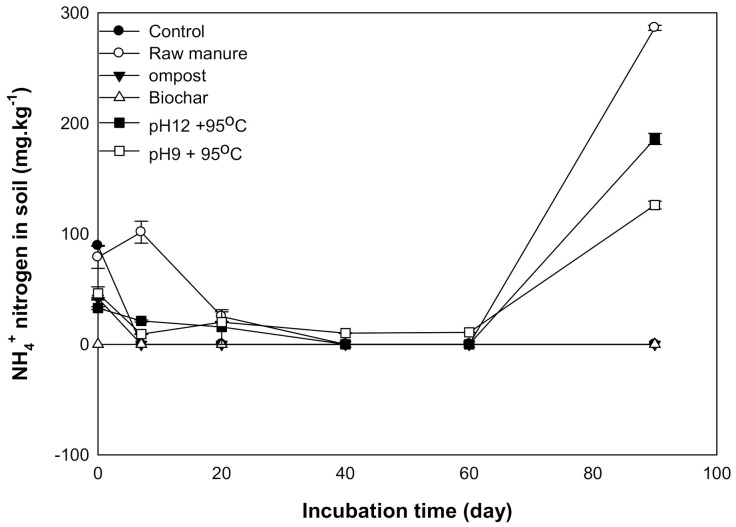
Available NH_4_^+^mg kg^−1^ affected by different organic amendments derived from cow manure at different incubation times.

The maximum NH_4_^+^ content was observed at 90 days, except for CMC and CMB (Figure 9). This seconded increase may be explained by the decomposition of the resistant material of CM and CMSAE such as protein, amino acids hemicelluloses [63] that produce NH_4_^+^.

### 3.3. Changes in Microbial Biomass Carbon (MBC)

**Figure 10:**
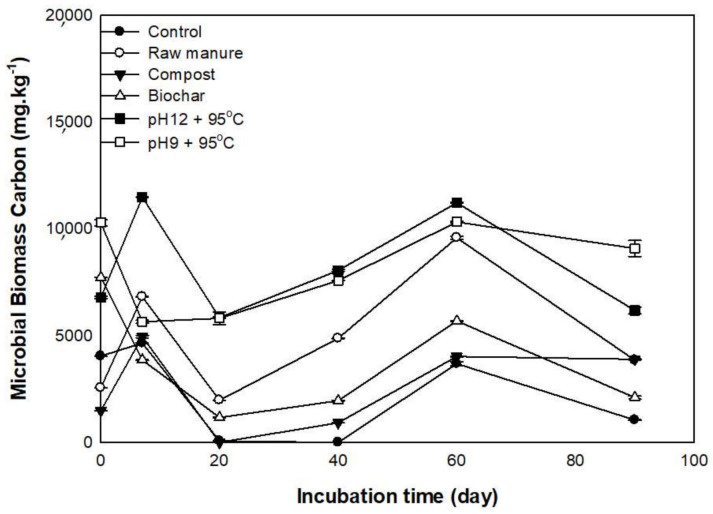
Effect ofdifferent organic amendments derived from cow manure and incubation time on soil microbial biomass carbon extracted with potassium sulfate.

Microbial biomass carbon in the soil is an indicator of the living component mass of the soil organic matter. The microbial biomass carbon plays a significant role in the availability and transformation of soil organic matter and plants’ nutrients uptake. Figure 10 shows the effect of organic materials and incubation time on microbial biomass carbon during 90 days. Most treatments showed an increase of MBC at seven days except CMB and CMSAE (pH 9). The CMSAE produced at pH 12 showed the highest MBC at seven days of incubation (11,481 ± 61 mg kg^−1^). The control, followed by CMB, showed the lowest soil MBC, compared to the rest of the treatments.

The increase of MBC indicated the presence of inorganic nitrogen and consequently high microbial activity, as suggested by [64], who showed the positive effect of organic materials on soil MBC. The soil microorganisms largely depend on easily degradable materials to increase their numbers and decompose more organic material. After 20 days of incubation, MBC content declined, probably due to the depletion of soluble organic carbon by microbes. The concentrated amino sugars, amino acids, proteins, and short-chain organic acids in CM [65] were reported to decompose fast in soil [66,67]. The low MBC in soils treated with biochar might be caused by the loss of many organic compounds that are consumed by microorganisms [25].

The second peak of MBC has been observed on day 60. A study also reported this on sheep dung decomposition. It was proposed that the first peak of CO_2_ emission (high MBC) is due to the decay of labile C from soil and easily degradable dung fractions. In contrast, decomposers attack more recalcitrant material in the second phase, hence increasing the MBC [68].

### 3.4. Changes in CO_2_ Efflux and Cumulative CO_2_

**Figure 11:**
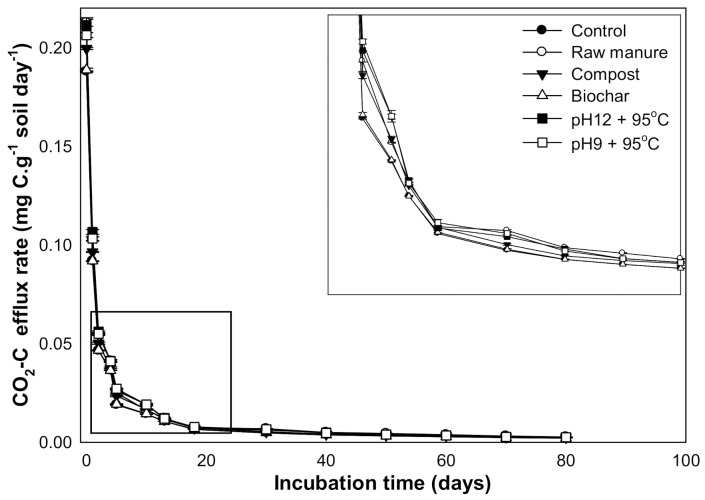
CO_2_ efflux rate (mg C/g soil/day) affected by different organic amendments derived from cow manure at different incubation times (marked box is magnified to show the trend).

The maximum CO_2_ efflux values were observed in the first seven days. After 20 days, CO_2_ efflux decreased with increasing incubation time. The high CO_2_ flux at the beginning might be due to the increased available nutrient content from organic manures and the increase in soil microorganisms’ activity. The soil CO_2_ efflux ranged from 0.231 to 0.001 g kg^−1^ soil. The highest CO_2_ fluxes were observed in CM-treated soils (0.231 g kg^−1^ soil) (Figure 11). In the short term, the addition of organic material provided additional substrates for the soil microorganisms and increased microorganism’s activity. This substrate may also relieve osmotic and pH stress on the soil microorganisms while improving chemical soil conditions [69].

**Figure 12:**
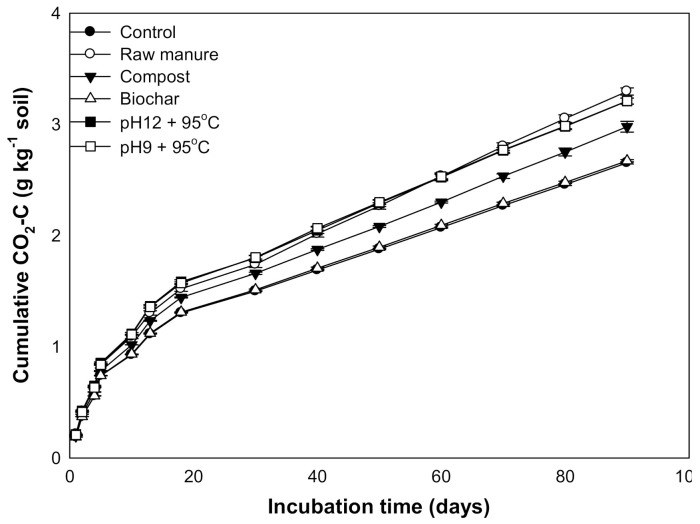
Cumulative CO_2_ efflux (g/kg soil) affected by different organic amendments derived from cow manure at different incubation times.

The results reveal that CMSAEs significantly increased the accumulated CO_2_ efflux (3.28 g kg soil^−1^) (Figure 12). This increase was almost similar to the rise in CO2 efflux by CM, while the CMB treated soils showed the smallest flux value (2.7 g kg soil^−1^). Our study thus clearly indicates that the CMSAE did not decrease the accumulative CO_2_ fluxes.

The low CO_2_ flux rate is possibly related to the low levels of MBC from 20 to 40 days. Other studies found that the microbial population densities in the initial days at organic matter addition were higher than the final days [70]. It was observed that the CMB and CMC proved to have less CO_2_ efflux, as compared to the CMSAE, due to the high C/N ratio of these materials, which was also indicated in the literature [71,72]. It was evident that the CMSAEs’ efflux values were higher than the other organic materials, including the CM, which is expected to have low CO_2_ flux.

## 4. Conclusions

In this experiment, a calcareous sandy loam soil was treated with different organic amendments derived from cow manure for 90 days. The results showed that the addition of different organic materials caused an increase in the available NO_3_^−^ nitrogen contents after 20 days of incubation time and decreased the available NH_4_^+^ concentration after 7 days from the start of incubation. MBC was significantly increased (*p* ≤ 0.05) as the incubation time increased. In the cow CMSAE produced at pH12 and T95 °C, CM and CMC showed the highest MBC; these values accounted for 11.481 ± 61 and 6815 ± 14 mg kg^−1^, respectively. At the same time, control and CMB reached their lowest MBC values after seven days.

Furthermore, the soil CO_2_ efflux ranged from 0.231 to 0.001 g kg^−1^ soil. The high CO_2_ efflux was observed in the raw CM and CMSAEs of pH12 and T95 °C; the values accounted for 0.231 and 0.211, respectively. Moreover, the results reveal that the CMSAEs at pH 12 and pH 9 with T95 °C decreased the accumulated CO_2_ efflux, while the CMB showed the smallest efflux value (19.8). Our study thus clearly indicates that the CMSAE did not reduce the accumulative CO_2_ efflux. Finally, the organic materials significantly affected the soil micronutrient cations (Fe, Cu, Zn, and Mn). The contents of available micronutrients were high at the beginning of incubation and then decreased over time.

## Data Availability

Not applicable.

## References

[1] Bouwman A., Van Der Hoek K. (1997). Scenarios of animal waste production and fertilizer use and associated ammonia emission for the developing countries. Atmos. Environ..

[2] Sutton M.A., Oenema O., Erisman J.W., Leip A., Van Grinsven H., Winiwarter W. (2011). Too much of a good thing. Nat. Cell Biol..

[3] Pinder R.W., Strader R.I., Davidson C., Adams P.J. (2004). A temporally and spatially resolved ammonia emission inventory for dairy cows in the United States. Atmos. Environ..

[4] Dubrovsky N.M., Hamilton P.A. (2010). Nutrients in the Nation? streams and groundwater: National Findings and Implications. Fact Sheet.

[5] FAO (2014). Agriculture, Forestry and Other Land Use Emissions by Sources and Removals by Sinks: 1990–2011 Analysis.

[6] Kuzyakov Y. (2006). Sources of CO_2_ efflux from soil and review of partitioning methods. Soil Biol. Biochem..

[7] Sallam A.S. (2002). Micronutrients status of Mollisols (southwestern mountainous region, Saudi Arabia). J. Saudi. Soc. Agr. Sci..

[8] Schjønning P., Thomsen I.K., Møberg J.P., de Jonge H., Kristensen K., Christensen B.T. (1999). Turnover of organic matter in differently textured soils: I. Physical characteristics of structurally disturbed and intact soils. Geoderma.

[9] Kowalska M., Güler H., Cocke D.L. (1994). Interactions of clay minerals with organic pollutants. Sci. Total. Environ..

[10] Risse L.M., Cabrera M.L., Franzluebbers A.J., Gaskin J.W., Gilley J.E., Killorn R., Radcliffe D.E., Tollner W.E., Zhang H. (2006). Land Application of Manure for Beneficial Reuse. Animal Agriculture and the Environment, National Center for Manure & Animal Waste Management White Papers.

[11] Yuan H., Lu T., Wang Y., Huang H., Chen Y. (2014). Influence of pyrolysis temperature and holding time on properties of biochar derived from medicinal herb (radix isatidis) residue and its effect on soil CO_2_ emission. J. Anal. Appl. Pyrolysis.

[12] Paré T., Dinel H., Schnitzer M., Dumontet S. (1998). Transformations of carbon and nitrogen during composting of animal manure and shredded paper. Biol. Fertil. Soils.

[13] Ameloot N., Sleutel S., Das K.C., Kanagaratnam J., De Neve S. (2015). Biochar amendment to soils with contrasting organic matter level: Effects on N mineralization and biological soil properties. GCB Bioenergy.

[14] Mohammed-Nour A., Al-Sewailem M., El-Naggar A.H. (2019). The Influence of Alkalization and Temperature on Ammonia Recovery from Cow Manure and the Chemical Properties of the Effluents. Sustainability.

[15] Van Vliet P.C.J., Reijs J.W., Bloem J., Dijkstra J., Goede R. (2007). Effects of Cow Diet on the Microbial Community and Organic Matter and Nitrogen Content of Feces. J. Dairy Sci..

[16] SAAF (2007). Space Image Atlas of the Kingdom of Saudi Arabia.

[17] Dere A.L., Stehouwer R.C. (2011). Labile and Stable Nitrogen and Carbon in Mine Soil Reclaimed with Manure-Based Amendments. Soil Sci. Soc. Am. J..

[18] Sparks D.L., Page A.L., Helmke P.A., Loeppert R.H. (2020). Methods of Soil Analysis, Part 3: Chemical Methods.

[19] Steiner C., Das K., Melear N., Lakly D. (2010). Reducing Nitrogen Loss during Poultry Litter Composting Using Biochar. J. Environ. Qual..

[20] Lehmann J. (2007). A handful of carbon. Nat. Cell Biol..

[21] Al-Wabel M.I., Al-Omran A., El-Naggar A.H., Nadeem M., Usman A.R. (2013). Pyrolysis temperature induced changes in characteristics and chemical composition of biochar produced from conocarpus wastes. Bioresour. Technol..

[22] Tomczyk A., Sokołowska Z., Boguta P. (2020). Biochar physicochemical properties: Pyrolysis temperature and feedstock kind effects. Rev. Environ. Sci. Bio. Technol..

[23] Xiang J., Liu D., Ding W., Yuan J., Lin Y. (2015). Effects of biochar on nitrous oxide and nitric oxide emissions from paddy field during the wheat growth season. J. Clean. Prod..

[24] Sigurnjak I., Brienza C., Snauwaert E., De Dobbelaere A., De Mey J., Vaneeckhaute C., Meers E. (2019). Production and performance of bio-based mineral fertilizers from agricultural waste using ammonia (stripping-) scrubbing technology. Waste Manag..

[25] Jindo K., Sanchez-Monedero M., Hernandez T., García C., Furukawa T., Matsumoto K., Sonoki T., Bastida F. (2012). Biochar influences the microbial community structure during manure composting with agricultural wastes. Sci. Total. Environ..

[26] Lima D., Santos S.M., Scherer H.W., Schneider R.J., Duarte A.C., Santos E., Esteves V.I. (2009). Effects of organic and inorganic amendments on soil organic matter properties. Geoderma.

[27] Estefan G. (2017). Methods of Soil, Plant, and Water Analysis: A Manual for the West Asia and North Africa Region.

[28] Creed J.T., Brockhoff C.A., Martin T.D. (1994). Method 2008: Determination of Trace Elements in Waters and Wastes by Inductively Coupled Plasma Mass Spectrometry.

[29] Kookana R.S., Sarmah A.K., Van Zwieten L., Krull E., Singh B. (2011). Biochar application to soil: Agronomic and environmental benefits and unintended consequences. Advances in Agronomy.

[30] Streubel J.D., Collins H.P., Garcia-Perez M., Tarara J., Granatstein D., Kruger C. (2011). Influence of Contrasting Biochar Types on Five Soils at Increasing Rates of Application. Soil Sci. Soc. Am. J..

[31] McCauley A., Jones C., Jacobsen J. (2009). Soil pH and organic matter. Nutr. Manag. Modul..

[32] Gramss G., Voigt K.-D., Bergmann H. (2004). Plant availability and leaching of (heavy) metals from ammonium-, calcium-, carbohydrate-, and citric acid-treated uranium-mine-dump soil. J. Plant Nutr. Soil Sci..

[33] Van Ranst E. (2006). Properties and Management of Soils in the Tropics.

[34] Hao X., Chang C. (2003). Does long-term heavy cattle manure application increase salinity of a clay loam soil in semi-arid southern Alberta?. Agric. Ecosyst. Environ..

[35] Goff J.P. (2006). Macromineral physiology and application to the feeding of the dairy cow for prevention of milk fever and other periparturient mineral disorders. Anim. Feed. Sci. Technol..

[36] Von Wandruszka R. (2006). Phosphorus retention in calcareous soils and the effect of organic matter on its mobility. Geochem. Trans..

[37] Delgado A., Madrid A., Kassem S., Andreu L., del Campillo M.C. (2002). Phosphorus fertilizer recovery from calcareous soils amended with humic and fulvic acids. Plant Soil.

[38] International Biochar Initiative (2013). Standardized Product Definition and Product Testing Guidelines for Biochar That is Used in Soil.

[39] García-Mina J., Antolín M.C., Sánchez-Díaz M. (2004). Metal-humic complexes and plant micronutrient uptake: A study based on different plant species cultivated in diverse soil types. Plant Soil.

[40] Colombo C., Palumbo G., Sellitto V.M., Rizzardo C., Tomasi N., Pinton R., Cesco S. (2012). Characteristics of Insoluble, High Molecular Weight Iron-Humic Substances used as Plant Iron Sources. Soil Sci. Soc. Am. J..

[41] Abadía J., Vázquez S., Rellán-Álvarez R., El-Jendoubi H., Abadía A., Álvarez-Fernández A., López-Millán A.F. (2011). Towards a knowledge-based correction of iron chlorosis. Plant Physiol. Biochem..

[42] Bavaresco L., Poni S. (2003). Effect of Calcareous Soil on Photosynthesis Rate, Mineral Nutrition, and Source-Sink Ratio of Table Grape. J. Plant Nutr..

[43] Zeng W., Zeng M., Zhou H., Li H.G., Xu Q.X., Li F. (2014). The effects of soil pH on tobacco growth. J. Chem. Pharm. Res..

[44] Aaltonen H., Palviainen M., Zhou X., Köster E., Berninger F., Pumpanen J., Köster K. (2019). Temperature sensitivity of soil organic matter decomposition after forest fire in Canadian permafrost region. J. Environ. Manag..

[45] Smith S.R. (2009). A critical review of the bioavailability and impacts of heavy metals in municipal solid waste composts compared to sewage sludge. Environ. Int..

[46] Ingelmo F., Molina M.J., Soriano M.D., Gallardo A., Lapeña L. (2012). 46 bioavailability of heavy metals in a sludge based compost. J. Environ. Manag..

[47] Grunditz C., Dalhammar G. (2001). Development of nitrification inhibition assays using pure cultures of nitrosomonas and nitrobacter. Water Res..

[48] Vadivelu V., Keller J., Yuan Z. (2007). Free ammonia and free nitrous acid inhibition on the anabolic and catabolic processes of Nitrosomonas and Nitrobacter. Water Sci. Technol..

[49] Agehara S., Warncke D. (2005). Soil moisture and temperature effects on nitrogen release from organic nitrogen sources. Soil Sci. Soc. Am. J..

[50] Kampschreur M.J., Tan N.C.G., Kleerebezem R., Picioreanu C., Jetten M.S.M., Van Loosdrecht M.C.M. (2007). Effect of Dynamic Process Conditions on Nitrogen Oxides Emission from a Nitrifying Culture. Environ. Sci. Technol..

[51] Usman A., Almaroai Y.A., Ahmad M., Vithanage M., Ok Y.S. (2013). Toxicity of synthetic chelators and metal availability in poultry manure amended Cd, Pb and as contaminated agricultural soil. J. Hazard. Mater..

[52] Preusch P., Adler P., Sikora L., Tworkoski T. (2002). Nitrogen and phosphorus availability in composted and uncom-posted poultry litter. J. Environ. Qual..

[53] Pichtel J. (1990). Microbial respiration in fly ash/sewage sludge-amended soils. Environ. Pollut..

[54] Paul J.W., Beauchamp E.G. (1994). Short-term nitrogen dynamics in soil amended with fresh and composted cattle manures. Can. J. Soil Sci..

[55] Whalen J.K., Chang C., Clayton G.W., Carefoot J.P. (2000). Cattle Manure Amendments Can Increase the pH of Acid Soils. Soil Sci. Soc. Am. J..

[56] Cai Y., He Y., He K., Gao H., Ren M., Qu G. (2019). Degradation mechanism of lignocellulose in dairy cattle manure with the addition of calcium oxide and superphosphate. Environ. Sci. Pollut. Res..

[57] Hampel J.J., McCarthy M.J., Gardner W.S., Zhang L., Xu H., Zhu G., Newell S.E. (2018). Nitrification and ammonium dynamics in Taihu Lake, China: Seasonal competition for ammonium between nitrifiers and cyanobacteria. Biogeosciences.

[58] Nyawade S.O., Karanja N.N., Gachene C.K., Gitari H.I., Schulte-Geldermann E., Parker M.L. (2019). Short-term dynamics of soil organic matter fractions and microbial activity in smallholder potato-legume intercropping systems. Appl. Soil Ecol..

[59] Haroon B., Abbasi A.M., An P., Pervez A., Irshad M., Faridullah (2018). Chemical Characterization of Cow Manure and Poultry Manure after Composting with Privet and Cypress Residues. Commun. Soil Sci. Plant Anal..

[60] Wang X.-L., Park S.-H., Lee B.-R., Jeong K.-H., Kim T.-H. (2018). Changes in Nitrogen Mineralization as Affected by Soil Temperature and Moisture. J. Korean Soc. Grassl. Forage Sci..

[61] Kirckner M., Wollum A., King L. (1993). Soil microbial populations and activities in reduced chemical input agroeco-system. J. Soil Sci. Am. Soc..

[62] Sharifnia S., Khadivi M.A., Shojaeimehr T., Shavisi Y. (2016). Characterization, isotherm and kinetic studies for am-monium ion adsorption by light expanded clay aggregate (LECA). J. Saudi Chem. Soc..

[63] Narita H., Zavala M.A.L., Iwai K., Ito R., Funamizu N. (2005). Transformation and characterisation of dissolved organic matter during the thermophilic aerobic biodegradation of faeces. Water Res..

[64] Jara-Samaniego J., Pérez-Murcia M., Bustamante M., Pérez-Espinosa A., Paredes C., López M., López-Lluch D., Gavilanes-Terán I., Moral R. (2017). Composting as sustainable strategy for municipal solid waste management in the Chimborazo Region, Ecuador: Suitability of the obtained composts for seedling production. J. Clean. Prod..

[65] Morvan T., Nicolardot B. (2009). Role of organic fractions on C decomposition and N mineralization of animal wastes in soil. Biol. Fertil. Soils.

[66] Glanville H., Rousk J., Golyshin P., Jones D. (2012). Mineralization of low molecular weight carbon substrates in soil solution under laboratory and field conditions. Soil Biol. Biochem..

[67] Kögel-Knabner I. (2002). The macromolecular organic composition of plant and microbial residues as inputs to soil organic matter. Soil Biol. Biochem..

[68] Ma X., Ambus P., Wang S., Wang Y., Wang C. (2013). Priming of Soil Carbon Decomposition in Two Inner Mongolia Grassland Soils following Sheep Dung Addition: A Study Using 13C Natural Abundance Approach. PLoS ONE.

[69] Chander K., Goyal S., Kapoor K. (1994). Effect of sodic water irrigation and farmyard manure application on soil mi-crobial biomass and microbial activity. Appl. Soil Ecol..

[70] Martín-Olmedo P., Rees R.M. (1999). Short-term N availability in response to dissolved-organic-carbon from poultry manure, alone or in combination with cellulose. Biol. Fertil. Soils.

[71] Cayuela M.L., Sánchez-Monedero M.A., Roig A., Hanley K., Enders A., Lehmann J. (2013). Biochar and denitrification in soils: When, how much and why does biochar reduce N_2_O emissions?. Sci. Rep..

[72] Huang G., Wong J., Wu Q., Nagar B. (2004). Effect of C/N on composting of pig manure with sawdust. Waste Manag..

